# Anticancer Activity of Nanoparticles Based on PLGA and its Co-polymer: *In-vitro* Evaluation 

**Published:** 2013

**Authors:** Issa Amjadi, Mohammad Rabiee, Motahare-Sadat Hosseini

**Affiliations:** a ***Biomaterial Group, Faculty of Biomedical Engineering (Center of Excellence), Amirkabir University of Technology, P. O. Box: 15875-4413, Tehran, Iran.***; b ***Polymer Group, Polymer Engineering Department (Center of Excellence), Amirkabir University of Technology, P. O. Box: 15875-4413, Tehran, Iran. ***

**Keywords:** Poly(lactic-co-glycolic acid), Doxorubicin, Nanoparticle, Sustained release, Antitumor activity

## Abstract

Attempts have been made to prepare nanoparticles based on poly(lactic-co-glycolic acid) (PLGA) and doxorubicin. Biological evaluation and physio-chemical characterizations were performed to elucidate the effects of initial drug loading and polymer composition on nanoparticle properties and its antitumor activity.

PLGA nanoparticles were formulated by sonication method. Lactide/glycolide ratio and doxorubicin amounts have been tailored. Fourier transform infrared spectroscopy (FTIR) and differential scanning calorimetry (DSC) were employed to identify the presence of doxorubicin within nanospheres. The in vitro release studies were performed to determine the initial ant net release rates over 24 h and 20 days, respectively. Furthermore, cytotoxicity assay was measured to evaluate therapeutic potency of doxorubicin-loaded nanoparticles.

Spectroscopy and thermal results showed that doxorubicin was loaded into the particles successfully. It was observed that lactide/glycolide content of PLGA nanoparticles containing doxorubicin has more prominent role in tuning particle characteristics. Doxorubicin release profiles from PLGA 75 nanospheres demonstrated that the cumulative release rate increased slightly and higher initial burst was detected in comparison to PLGA 50 nanoparticles. MTT data revealed doxorubicin induced antitumor activity was enhanced by encapsulation process, and increasing drug loading and glycolide portion. The results led to the conclusion that by controlling the drug loading and the polymer hydrophilicity, we can adjust the drug targeting and blood clearance, which may play a more prominent role for application in chemotherapy.

## Introduction

Cancer is a disease which refers to a group of tumor types formed by uncontrolled proliferation of abnormal cells. If the rapid growth is not controlled by normal cells, it can lead to pathological outcome like tissue fractures. Cancer is the second cause of death in the developed countries and is steadily increasing in both industrialized and developing countries (1). The most recent global statistics estimate that the number of people suffering from cancer will increase to more than 15 million in 2020 (2). Chemotherapy is a conventional approach with the major potential to cure localized and metastasized cancers (3). In this way anticancer drugs are infused through blood vessels and after an initial rapid increase in the drug concentration, the intravenous injections cause the drug decomposition in the blood (4). On the other hand, most kinds of anticancer agents do not distinguish between cancer cells and the normal ones (5), resulting in adverse reactions in tissue organs (3). Drug delivery systems can reduce these inefficiencies via encapsulation methods, which decrease lesion of the toxic drug, and protect the drug before reaching the target cells (6). Due to lipophilic characteristics of cells membrane, only hydrophobic low molecular weight drugs can cross the membrane (7). Therefore, action of polar compounds is limited (7).

Bionanotechnology seem to be a more promising approach than conventional methods. Because the nanoscopic particles have great potentials for anticancer drugs encapsulation (3), drug targeting and mediators in cancer magnetic hyperthermia (7, 8), as well as unique functional characteristics such as small size, high stability, low toxicity (3), easy purification and sterilization, *etc *(1). Moreover, human cells’ diameter is roughly btween10 and 20 μm and the size of cell organelles range from nanometers to a few hundred nanometers (1). Thus, nanoparticles can overcome physiological barriers and readily interact with intracellular compartments without any additional surgery (3).

Furthermore, poor lymph flow improves enhanced permeability and retention effect, and makes it reside in the tumor for a considerably long period of time (9). Poly(L-lactide-co-glycolide) (PLGA) has been approved by FDA for target drug delivery using NPs and tissue engineering. Several advantages can be mentioned for PLGA, such as alteration of mole fraction of lactide and glycolide, and modification of molecular weight which regulates degradation period and water-induced degradation which in turn, produces biocompatible by-products which are further eliminated through metabolism (10, 11). 

The aim of this research is to prepare an adjustable drug delivery system with optimized release rate. To attain this goal, doxorubicin as an appropriate anticancer with wide application in chemotherapy was loaded in biodegradable PLGA (lactide/glycolide; 50:50 and 75:25) and effects of alteration on nanoparticle properties and antitumor activity were studied.

## Experimental

*Materials*

Doxorubicin hydrochloride (Sigma, USA) was employed to encapsulate in NPs. and PLGA 50:50 and 75:25 manufactured by Boehringer Ingelheim (Ingelheim, Germany) was used as the base materials. Dichloromethane (DCM) and poly(vinyl alcohol) (PVA; M_n_= 72000 g/mol and 99.8% hydrolysis) were purchased from Merck (Germany).

*Nanoparticles preparation*

NPs were prepared by Double emulsion technique because of high water solubility of Dox. The drug solution was added dropwise via a syringe into DCM (2.5 mL) and PLGA (50 mg) by sonicating for two min in an ice bath to form W_1_/O emulsion. PVA (3% w/v) was added and sonicated for 2 min (secondary emulsion; W_1_/O/W_2_). The final emulsion was continually stirred for 18 h to evaporate DCM. The particles were gathered by centrifugation at 1500 rpm, rinsed in deionized water 3 times, and then lyophilized at -75°C and 0.03 Pa (12).

*FTIR characterization*

The effects of encapsulation process on the chemical group and the interaction between the components was studied by performing a fourier transform infrared spectroscopy (FTIR) model Bruker Equinox 55 (USA). The FTIR spectra ranging between 500 cm^-1^ and 4000 cm^-1^ were obtained by mixing samples with KBr powder (Infrared grade). 

*Thermal characterization*

The thermal properties of the samples were determined using a differential scanning calorimetry (DSC; 200 F3 Maia^®^, Netzsch, Germany). The test was conducted according to ISO/IEC 17025 standard. The samples were heated at a rate of 10 °C.min^-1^ from room temperature to 350 °C.

*Particle size analysis and Zeta potential*

The distribution of the particles was evaluated by measuring the polydispersity index (PDI) using a particle size analyzer model Nano ZS (Malvern Instrument Ltd, UK). When the PDI values varied between 0.01 and 0.7, the particles would have narrow distributions. The high values of PDI (PDI > 0.7) indicated very broad distributions (8). Zeta potential was determined by sonicating the samples in 1 mL of distilled water, and then subjecting to dynamic light scattering.

*Entrapment efficiency and in-vitro release study*

**Figure 1:**
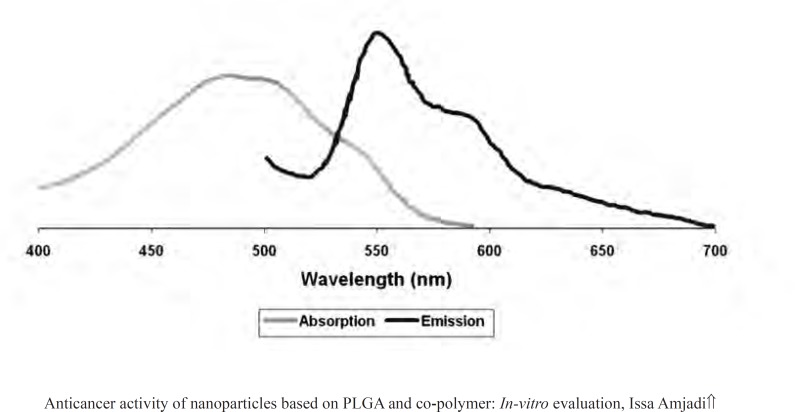
Absorption and emission spectra of Dox

Centrifugation method was performed to determine the encapsulation efficiency (EE) (13). The particles were precipitated, and then a certain proportion of fresh particles were dissolved in acetonitrile. The entrapment efficiency was measured by using a fluorescent spectrometer model Perkin Elmer LS55 (USA). The drug was detected at 470 nm (excitation) and 550 nm (emission) (Figure 1) (14). The EE parameter was calculated as followed (15): 

$\mathrm{EE}-\frac{W_{t}-W_{d}}{W_{1}}$

Where W_t_ and W_d_ describes the total drug added and the drug extracted into the supernatant, respectively.

In addition, the drug release from PLGA particles was determined in phosphate buffer saline (PBS; pH 7.4, o.15 M) shaken moderately.

*Morphological evaluation*

The morphology of the synthesized samples was assessed by performing a scanning electron microscopy (SEM) model Seron Technologies AIS 2100 (Korea) on the gold coated surface of samples at 15 KV.

*Cell culture and cytotoxicity evaluation*

Mouse fibrosarcoma cells (L929) were cultured in RPMI (Gibco, USA) containing 10% FBS (Seromed, Germany) and incubated in 5% CO_2_ at 37°C. To determine the Median lethal concentration (LC_50_) of doxorubicin, various concentrations of drug were poured into the tissue culture polystyrene (TPS) 96-well plate (5×10^3^ cells/well) and then incubated at 37 °C and 5% CO_2_ for 24 h. The TPS wells without drug were used as control. At the end of the treatment period, cell viability was measured by performing dimethylthiazol diphenyl tetrazolium bromide (MTT; Sigma, USA) assay. Briefly, the medium was removed and 100 μm of MTT (0.5 mg/mL) was added. The formazan crystals were then dissolved in dimethyl sulfoxide (DMSO; 100 μL, Sigma, USA). The plate was then incubated at 37 °C for 4 h. The optical density (OD) was read with multiwell microplate reader (ELIZA reader, Organon Teknika, Netherlands) at 570 nm. Moreover, cell proliferation was also measured to explore the effect of encapsulation process, polymer composition, and drug loading on doxorubicin induced cytotoxicity over three days (7). 

## Results

*Doxorubicin encapsulation studies*

**Figure 2:**
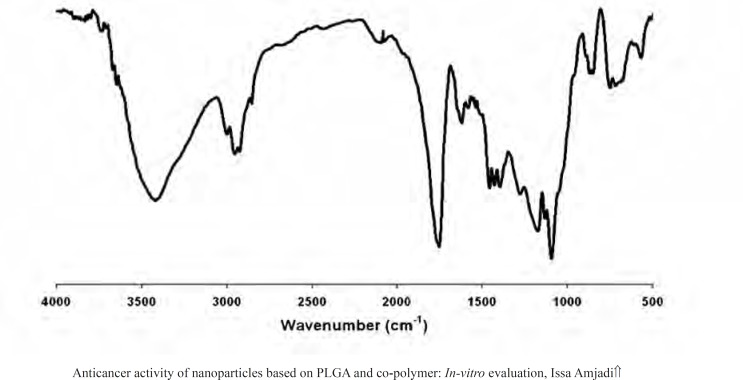
FTIR-transmission spectrum of Dox loaded-PLGA NPs

The presence of the drug within the nanoparticles was evaluated by FTIR. The FTIR spectra for doxorubicin-loaded nanoparticles were presented in Figure 2. The strong and wide peak at 3420 cm^-1^ correlated to the hydroxyl and amine groups overlapping each other exhibited the presence of the drug molecule in the nanoparticles, meaning that the drug was encapsulated in PLGA 50 nanoparticles.

**Figure 3:**
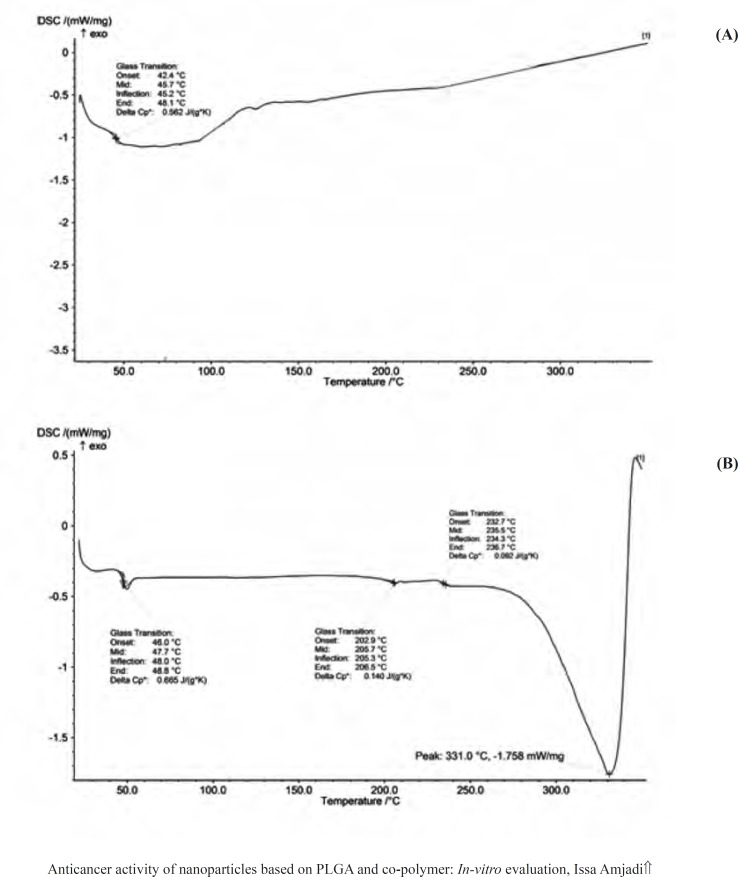
DSC thermogram of (a) empty NPs and (b) Dox-loaded NPs

DSC curves provided information on the thermal transformations in the nanoparticles and the state of the drug after encapsulation process. Figure 3 showed DSC thermogram of the empty PLGA particles and corresponding nanoparticles containing doxorubicin. The glass transition temperature (Tg) values of the empty and encapsulated PLGA 50 were relatively about 48 °C, which were in agreement with the reported T_g _of PLGA 50 in the literatures (between 40 °C and 60 °C). Doxorubicin had a melting point (T_m_) about 230 °C, resulting in an endothermic peak in the DSC curve and giving the evidence for crystal structures of the drug. The absence of native doxorubicin Tm peak in the DSC curve of doxorubicin-loaded PLGA 50 particles represented that the drug in the nanoparticles were converted to amorphous phase. In other words, encapsulation process disrupted the native doxorubicin crystals, indicating that the encapsulation process was appreciable.

*Effect of initial drug loading and copolymer composition on the particle properties*

**Figure 4:**
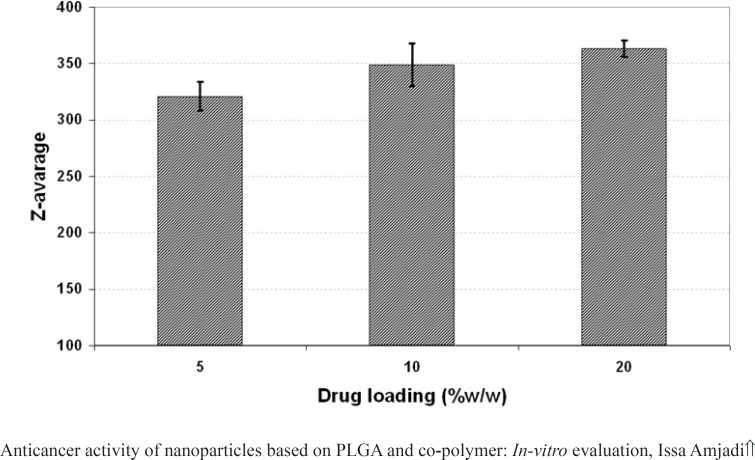
Effect of drug loading on the size of PLGA NPs

**Figure 5:**
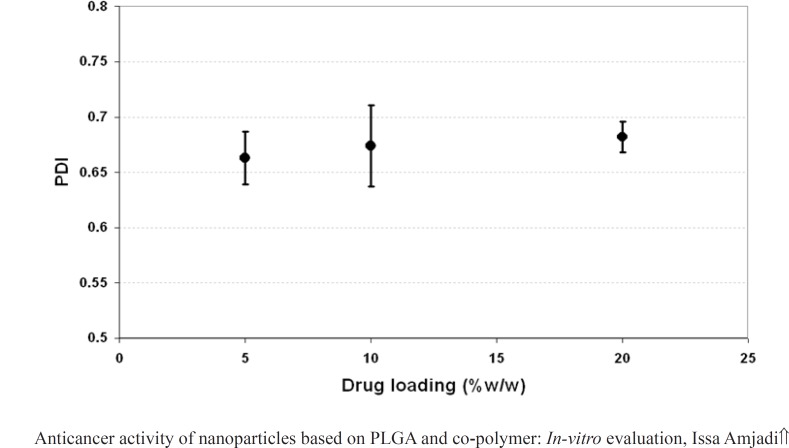
Effect of drug loading on the PDI of Dox- loaded PLGA NPs

**Figure 6:**
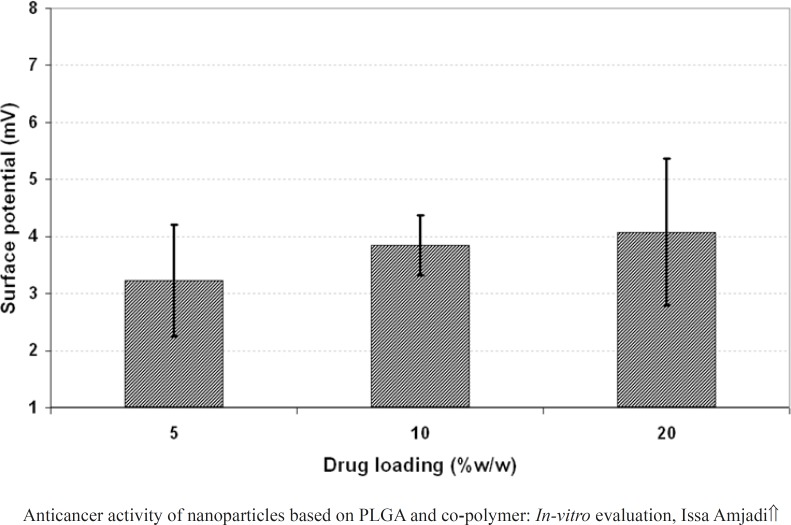
Effect of polymer characteristics on the surface potential

The effect of drug loading (5, 10 and 20% w/w of PLGA 50) on the properties of the nanoparticles including particle size, PDI and surface potential was presented in Figure 4, 5, and 6, respectively. As it could be observed in figure 4 and 5, drug loading had no significant effect on the particle size and surface potential. However, a slight increase in particle size and surface potential versus drug loading. The size of 5, 10, and 20 percent of doxorubicin loaded nanoparticles were 320.5, 363.1, and 370.8, respectively. It is apparent that the increase in the particle size could be well pertained to high levels of the drug. Figure 6 illustrated that surface potential was positive for all the samples due to the presence of positively charged amine group in the drug structure. Therefore, loading of 10% gave particles with optimum diameter and polydispersity.

Table 1 summarizes the measured values of particle size and PDI for doxorubicin-loaded PLGA 50 and 75 nanoparticles. Results indicated that the size of the particles relatively seemed to remain constant as the PLGA composition was changed. In addition, PDI data revealed that PLGA 75 nanoparticles had the uniform diameter than PLGA 50 nanoparticles (PDI < 0.7). 

The drug entrapment efficiency demonstrated in Table 2. The value of entrapment efficiency for the samples decreased as the lactide to glycolide content in the copolymer composition increased. Therefore, there was a molecular dispersion of doxorubicin within PLGA 75 matrix. 

**Figure 7:**
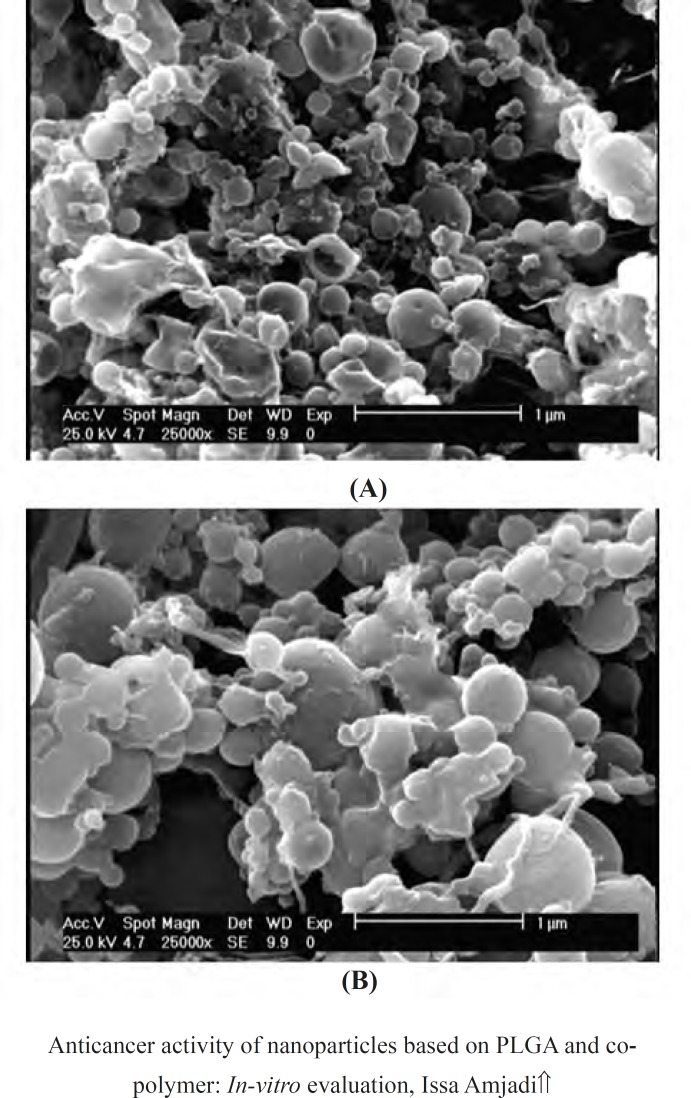
SEM micrographs of (a) Dox-loaded PLGA 50:50 and (b) Dox loaded-PLGA 75:25

SEM images of the nanoparticles based on doxorubicin and PLGA were showed in Figure 7. The particles had smooth surfaces and spherical topography with some agglomerations and less and more narrow size distributions which could be related to the data measured by size analyzer. 

**Table 1:** Physical characteristics for doxorubicin loaded PLGA 50 and 75 nanoparticles

**Lactide (%) **	**Particle size (nm) **	**PDI **	**Surface charge (mV) **
50	363.1	0.663	3.85±0.53
75	361.4	0.417	1.29±0.08

**Table 2:** Entrapment efficiency data for PLGA 50 nanoparticles and its corresponding copolymer with 75% lactide content

**Lactide (%) **	**Entrapment effeciency (%) **
**50 **	48.37
**75 **	38.65

*In-vitro release evaluation*

**Figure 8:**
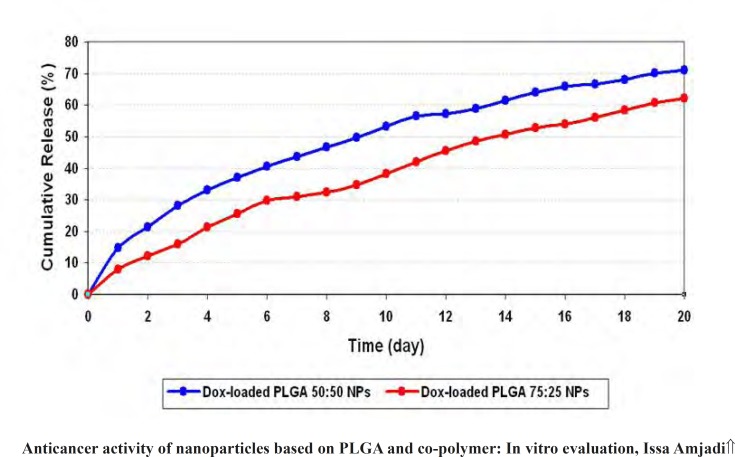
*In-vitro *release of Dox-loaded PLGA 50:50 NPs (blue) and Dox-loaded PLGA 75:25 NPs (red) in phosphate buffer pH 7.4

Release profiles of doxorubicin from PLGA 50 and PLGA 75 nanoparticles in PBS were determined. Cumulative release curves were shown in figure 8. The drug release from PLGA 50 and PLGA 75 nanoparticles in the first day was about 7.91% and 14.70% of total doxorubicin, respectively. Then, the release rate increased slowly without any burst effect. The cumulative curves of PLGA 50 and PLGA 75 nanoparticles displayed release of 70.98% and 62.22% over 20 days. 

*The potential of nanoparticles in doxorubicin induced toxicity*

**Figure 9:**
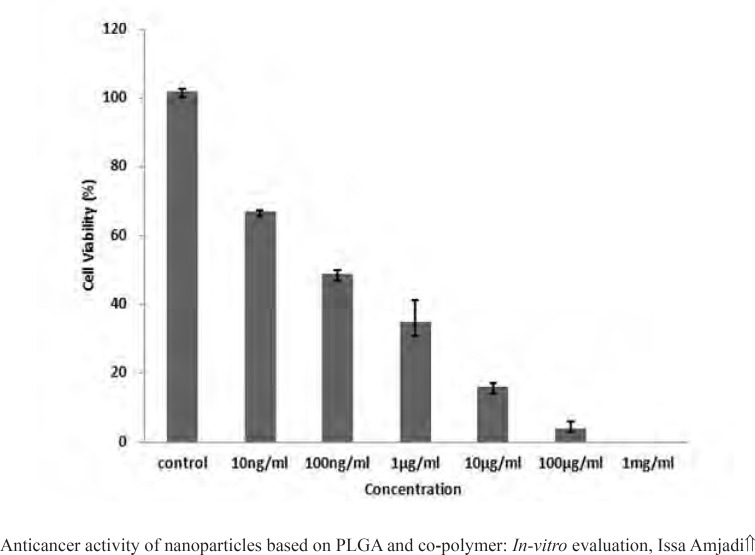
Viability of L929 cells according to MTT test with different concentrations of Dox solutions.

**Figure 10:**
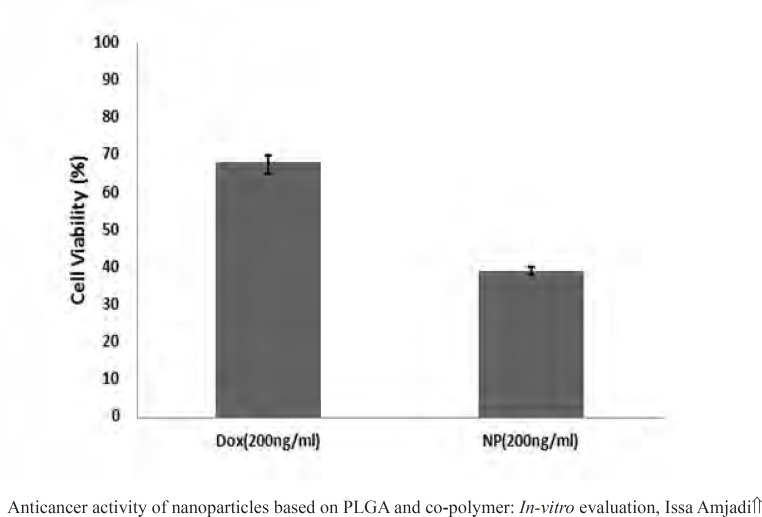
Proliferation percent of cells treated by Dox and NPs at twice concentration of the LC_50_.

MTT assays have been proved practical for determination of sublethal doses of drugs, potential therapeutic agents applied for chemotherapy, and succeeding detailed studies of the pathways by which the chosen anticancer drug acts. Figure 9 presented the cancer cell line exposed to the various doxorubicin concentrations. A reduction in cells viability was observed in the MTT data indicating that anticancer and antiproliferative activities of doxorubicin were concentration-dependent. Moreover, the LC_50_ value of the drug was 100 ng.mL^-1^. Then to evaluate in vitro antitumor activity of the nanoparticles, doxorubicin (200 ng.mL^-1^) was encapsulated into the PLGA 50. The cell viability for the nanoparticles at the concentration higher than the LC_50_ value over 24 h has been calculated to be 39.12%, meaning that the same drug content entrapped within the nanoparticles were found to be more toxic than the free drug (Figure 10). 

**Figure 11:**
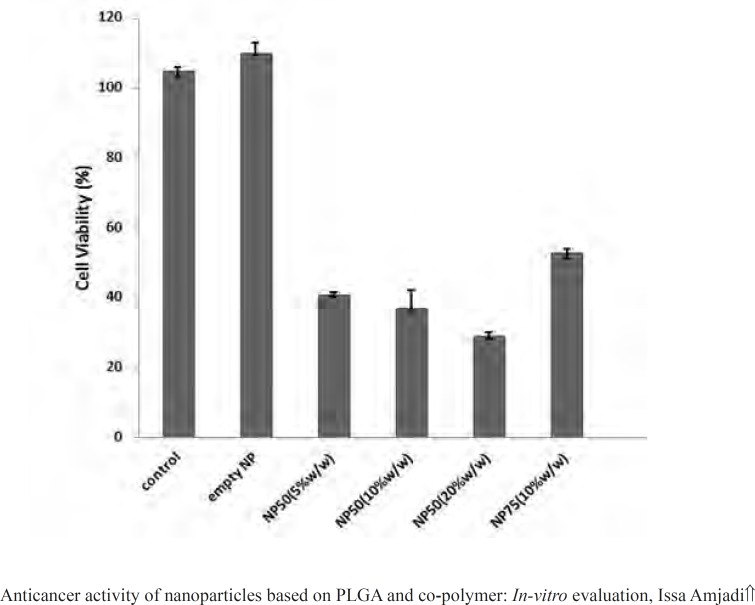
Cellular response to the different amounts of drug loading and PLGA compositions

Figure 11 illustrated proliferation rate of L929 cells treated by empty PLGA 50 and 75 nanoparticle, PLGA 75 containing doxorubicin (10% w/w of PLGA) as well as PLGA 50 loaded by various initial drug loading (5, 10, and 20% w/w of PLGA). 

## Discussion

Most drugs which have been used in chemotherapy have rapid blood clearance, low tumor selectivity, and heavy poisonous side effects for normal tissues (16). Nanoparticless reduce these side effects (17), improve their distribution in the body (10), increase their specificity (11), prolong activity (10), and improve their in vivo degradation resistance (18). In this study PLGA particles were developed specifically for sustained release of the water-soluble drug. According to the outline of the International Conference on Harmonization (12), dichloromethane fell under class two solvents which were generally considered as solvents with lower risk to human health. Hence, its use was permitted. During drug formulation, the presence of additional water in aqueous phases and stirring overnight led DCM to diffuse and subsequently evaporate (16). Remnants of organic solvent has negligible amount which is not hazardous. 

The experimental results showed low positive surface charge for the PLGA 75 nanoparticles leading to some agglomeration and instability of the nanospheres. For targeted drug delivery systems, some factors including hydrophilicity and surface charge play leading roles to improve blood circulation time and anticancer drug concentration in the tumor (19, 20). On the other hand, since glycolide groups are more hydrophilic than lactide group, PLGA 75 nanospheres were more lipophilic than PLGA 50 ones, meaning that as lactide contents increased the nanoparticles became less hydrophilic which resulted in reducing the rate of blood clearance and rapid removal from the blood stream by the immune system (21). 

During the fabrication of doxorubicin loaded particles, it was found that a higher lactide/glycolide ratio decreased the values of entrapment efficacy of the PLGA 75 particles. In other words, doxorubicin dispersed in the PLGA 75 particles, because there has been a low molecular affinity between the high water soluble drug and the lipophilic carrier, decreasing the efficient interactions of doxorubicin and PLGA 75 (22, 23). The size, zeta potential and PDI seemed to be unaffected by change in drug loading values and lactide/ glycolide ratio. It was well pertained to the same stabilizer concentration (PVA) and the encapsulation process (sonication method). PVA is a cationic surfactant and greatly used as stabilizer in drug formulation. The presence of PVA as a stabilizer has prominent affects on surface charge of particles. Charge differences between positive nanoparticle and negatively charged mucosa cause electrostatic interactions and increase intestinal uptake of the nanospheres containing antitumor drugs (12). Doxorubicin release profiles from PLGA 75 nanospheres demonstrated that the cumulative release rate increased slightly and higher initial burst was detected in comparison to PLGA 50 nanoparticles. In terms of *in-vitro *drug release, two mechanisms were involved: diffusion and degradation (24). Initial release of drug into the medium is more related to the migration and rapid motion of drug molecules on the surface of nanoparticles and depended on drug solubility and polymer hydrophilicity (25). Therefore, doxorubicin molecules entrapped among PLGA 75 chains preferred to migrate into the medium as a function of solubility. Subsequent net release rate is correlated to the degradation rate of PLGA bulk. PLGA hydrolyzes into nontoxic low molecular weight compounds. Thus, the drug type and copolymer composition determined the rate of PLGA degradation and then cumulative doxorubicin release rate. Doxorubicin and glycolide groups have been shown high affinity to water molecules. In other words, glycolic acid portion and water solubility of doxorubicin are crucial parameters in altering the drug release profile. 

The viability of L929 cells treated by doxorubicin-loaded nanospheres reduced significantly in comparison to the empty particles and untreated cells. In addition, these results demonstrated that cells response to the nanoparticles was dependent on the various amounts of the drug loaded into the particles and the net release rate. An increase in the cell toxicity suggested that nanospheres did not aggregate in cell culture medium and cross the cellular membrane.

## Conclusion

In this work, we employed an easy method to formulate the doxorubicin-loaded PLGA nanoparticles. The nanoparticles properties could be tuned by adjusting the drug amounts and the polymer composition. It is also appreciable to reveal that glycolide content and sonication method have a significant effect on the drug solubility and cumulative release rate. Therefore, by controlling the drug loading and the polymer hydrophilicity, we can adjust the drug targeting and blood clearance, which may play a more prominent role for application in chemotherapy. 
